# Glycosylation of anthocyanins enhances the apoptosis of colon cancer cells by handicapping energy metabolism

**DOI:** 10.1186/s12906-020-03096-y

**Published:** 2020-10-15

**Authors:** Nan Jing, Jiaxing Song, Zheng Liu, Luoyang Wang, Guoqiang Jiang

**Affiliations:** 1 Key Lab of Industrial Biocatalysis, Ministry of Education, Tsinghua University, Beijing, China; 2 Department of Chemical Engineering, Tsinghua University, Beijing, China

**Keywords:** Anthocyanin, Glycosides, Cell apoptosis, Glucose transporter

## Abstract

**Background:**

While anthocyanins are proven to be effective in inhibiting tumour cell proliferation, the underlying mechanisms remain unclear. This research aims to explore the glycosylation of anthocyanins in the tumour inhibitory effects and the potential mechanism.

**Methods:**

The tumour inhibitory effect on mouse colon cancer cells (MC38) was examined by MTT and flow cytometric analyses. The inhibitory pathway of anthocyanin was explored by assessment of tumour cell mitochondrial membrane potential (MMP), the caspase-3 and caspase-9 activity, as well as the cell energy metabolism in terms of the glucose uptake, the NAD^+^/NADH ratio and the ATP level.

**Results:**

We found that 500 μM bilberry anthocyanins extract (BAE) induced 48.1% mitochondrial damage, activated the downstream caspase cascade to form apoptotic bodies (caspase-3 activity increased by 169%, caspase-9 activity increased by 186%), and inhibited cell proliferation (survival rate: 55.97%, 24 h). In contrast, the same concentration of anthocyanidin (cyanidin) led to marginal mitochondrial damage (only 9.85%) and resulted in little inhibition of MC38 cells (survival rate: 86.84%, 24 h). For cells incubated with 500 μM BAE, reactive oxygen species (ROS) decreased by 53.8%, but the ratio of NAD^+^/NADH increased to 3.67, demonstrating that the mitochondrial damage was induced by blocking energy metabolism. Furthermore, cell energy metabolism is related to glucose uptake since the presence of 200 μM GLUT1 inhibitor substantially enhanced the inhibitory effects of cyanidin-3-O-glucoside (Cy-3-Glu) at 500 μM (survival rate: 51.08%, 24 h).

**Conclusions:**

The study suggested that the glycosides of anthocyanins might handicap glucose transport and inhibit energy metabolism, which, in turn, led to mitochondrial damage and apoptosis of tumour cells.

## Background

Anthocyanins are flavonoids found in various fruits and vegetables as natural plant pigments [1, 2] and are highly valued for their health-promoting attributes, such as promoting intestinal barrier function [3], prventing cardiovascular disease, and alleviating oxidative stress induced by ages, diabetes and inflammation [4, 5]. Anticancer effects have also been reported for anthocyanin extracts including bilberry, blueberry, cranberry and other berries [6–9]. Anthocyanin consist of the anthocyanidin aglycone plus one or more glycosides. To date, studies of the anticancer mechanisms of anthocyanins have focused on the anthocyanidin aglycone. The reported studies showed that anthocyanidin could affect signalling pathways related to proliferation and apoptosis of tumour cells [10], e.g., inhibiting the proinflammatory NF-κB pathway [11], targeting the PDK1-PI3K/Akt signalling pathway [12], enhancing the expression of p21WAF1 and suppressing the expression of cyclin A/B simultaneously [13].

However, the impact of the glycosides of anthocyanins on tumour inhibition has not been well clarified, and data on the influence on the bioactivity of glycosides were diverse or even controversial. On the one hand, O-glycosylation of flavonoids reduces their biological effects [14]. For example, the O-glycosylation of flavonoids dramatically diminished the inhibition on producing NO, expressing iNOS, and activating NF-κB in RAW264.7 macrophages and mouse microglial BV-2 cells [15]. Furthermore, in vitro experiments showed that flavonoid aglycones displayed better anticancer potency than their homologous glycosides [16, 17]. On the other hand, there are reports showing that glycosylation enhances the bioactivity of flavonoids, which may be attributed to the facilitated transmembrane delivery driven by glycoside binding to glucose transporters (GLUTs) on the cell membrane [18]. Zou et al. proved that these two inhibitors of glucose transporters (phloridzin and phloretin) could inhibit the absorption of cyanidin-3-O-glucoside (Cy-3-Glu) [19]. Manzano et al. reported that peonidin-3-O-glucose inhibits the activities of the glucose transporters which could further affect glucose absorption and transport by cells [20]. Alzaid et al. demonstrated that anthocyanin extracts from berries significantly reduced SGLT1 and GLUT2 expression by cells [21]. Despite the aforementioned efforts, the effects of glycosylation on the functions of anthocyanins, especially their antitumour function, remain indistinct.

The purpose of this research was to explore the glycosylation of anthocyanin on tumour cell inhibition and the related mechanism. We compared the tumour inhibitory efficiency of glycosylated anthocyanins on mouse colon cancer cells (MC38) with that of the anthocyanidin aglycone and found that glycosylation significantly increased the cytotoxicity of anthocyanins to tumour cells. Then, we demonstrated that the inhibitory pathway of anthocyanin was highly related to the energy metabolism of tumour cells, in which the glycosides of anthocyanins exerted a decisive influence. For the first time, to our knowledge, we proposed a potential mechanism by which glycosides of anthocyanins enhance tumour cell inhibition through energy metabolism and glucose transport.

## Methods

### Chemicals and reagents

The standardised bilberry (*Vaccinium myrtillus*) extracts (Mirtoselect®) were purchased from Indena SpA (Milan, Italy) which manufactured by their specific process to ensure the constant anthocyanin composition (36% w/w). The composition of the anthocyanin extracts was detected by HPLC-DAD-MS (Fig. S1, Table S1) with references [22]. The anthocyanin content was determined by comparing chromatographic peak areas with cyanidin-3-glucoside, an external standard. As shown in Table S1, 99.17% of the anthocyanins in bilberry are glycosylated. PBS, MTT, RIPA buffer, Hoechst 33258 and all assay kits except GOD-POD kit were acquired from Beyotime Institute of Biotechnology, Ltd. (Shanghai, China). GOD-POD kit was purchased from Beijing leagene biotechnology, Ltd. (Beijing, China). Dimethyl sulfoxide (DMSO), cyanidin-3-O-glucoside, cyanidin chloride and 5-fluorouracil (5-FU) were purchased from Sigma-Aldrich (St. Louis, MO). Chromatographic mobile phases were obtained from Fisher Scientific (Shanghai, China). Reagents for cell culture were obtained from Gibco (Grand Island, NY). WZB117 (glucose transporter 1 (GLUT1) inhibitor) was purchased from APExBIO (Houston, Texas). Other reagents were purchased from Sinopharm Chemical Reagent Co., Ltd. (Shanghai, China). All the other chemical reagents used in this study were of analytical grade and used as received.

### Cell culture

Dulbecco’s modified Eagle’s medium (DMEM) containing 10% foetal bovine serum and 1 × Penicillin-Streptomycin solution was used to culture the murine cancer cell line MC38 and the fibroblast cell line L929 (Cell Bank of the Type Culture Collection, Chinese Academy of Sciences). The cell lines were incubated under a humidified atmosphere (RH 85%) and 5% carbon dioxide at 37 °C [23, 24].

### Cell viability and apoptosis

The cells were seeded into a 96-well plate, and 200 μL of DMEM was added to each well to give a concentration of 1 × 10^5^ cells/well at 37 °C for 24 h. Then medium was removed and fresh DMEM containing four doses (100, 200, 500 and 1000 μM) of anthocyanin (ANC) of bilberry extract, anthocyanin standards (cyanidin-3-O-glucoside and cyanidin chloride) or GLUT1 inhibitor was added. The control groups did not contain ANC. The medium was removed after the cells incubated for 24, 48 and 72 h. The cell viability was determined by the MTT assay [25, 26]. The inhibitory effect on MC38 cells was determined based on the relative cell survival rate.

Apoptotic cells after treatment with ANC were quantified with an Annexin V-FITC detection kit. MC38 cells were seeded into a 6-well plate to give a concentration of approximately 2.5 × 10^6^ cells/well for 24 h of incubation, after which four doses (100, 200, 500 and 1000 μM) of ANC were added respectively and incubated at 37 °C for 48 h. DMEM was collected in an EP tube, and then adherent cells were washed with PBS. Afterwards, the adherent cells were digested with trypsin and resuspended in PBS buffer with the concentration of 1 × 10^6^ cell/mL approximately. Staining solution consisting of 195 μL of FITC-conjugated Annexin V binding buffer and 5 μL of Annexin V-FITC were added after centrifugation (1000 g, 5 min). After slightly vortexing, the mixture was incubated avoiding from light at room temperature (20–25 °C) for 15 min. The percentages of early and late apoptotic cells were determined by flow cytometry (FACS) analysis.

Cell apoptosis was also determined with confocal laser microscopy. Cells (1.5 × 10^4^) were seeded into a confocal petri dish and incubated for 24 h, after which DMEM containing 100, 200, 500, and 1000 μM ANC were added respectively and incubated for another 24 h. After that, DMEM containing ANC was removed and adherent cells were washed twice with PBS. One millilitre of the fluorescence staining reagent (1 × Hoechst 33258) was added. After incubation for 30 min, the staining reagent was removed, and the dish was washed with PBS twice, followed by observation with a fluorescence microscope at 450 nm.

### Mitochondrial membrane potential assay

Mitochondrial depolarisation in MC38 cells was determined by a JC-1 mitochondrial membrane potential (MMP) assay kit. Firstly, cells were incubated with four doses (100, 200, 500 and 1000 μM) of ANC or anthocyanin standards for 24 h in 6-well plates. Next, an EP tube was used to collect cell culture medium. Afterwards, adherent cells were washed with PBS and digested with trypsin. Then, approximately 1 × 10^6^ cells were resuspended in one milliliter of PBS and one milliliter of JC-1 staining solution (5 μg/mL) was added. Cells were incubated at 37 °C for 20 min and washed twice with PBS. The MMP was monitored by FACS analysis at 488 nm. Mitochondrial depolarisation is presented by the ratio of fluorescence intensity.

### The change of caspase activity

Caspase-3 and caspase-9 activity was determined by caspase activity assay kits. Briefly, cells (2.5 × 10^6^) were seeded into a 6-well plate and incubated for 24 h. Culture media containing four doses (100, 200, 500 and 1000 μM) of ANC were added separately for 48 h of incubation. Then, an EP tube was used to collect cell culture medium. Next, adherent cells were washed with PBS and digested with trypsin, following by a reaction in an ice bath for 15 min with addition of 100 μL of cold cell lysis buffer. The lysed cells were transferred to a tube (cell lysate). Cell lysates were collected for centrifugation (20,000 g, 15 min) at 4 °C, yielding supernatants subjected to determination of the protein concentration with a total protein quantification kit. Afterwards, approximately 20 μg of total protein was taken to determine caspase activity as operating instruction described. The caspase activity was exhibited as a percentage of the enzyme activity compared with the control.

### Assay of intracellular reactive oxygen species (ROS)

The intracellular level of ROS was monitored by detecting oxidative conversion of 2′,7′-dichlorofluorescein diacetate (DCFH-DA) into fluorescent dichlorofluorescein (DCF) with flow cytometry. Cells were washed with PBS, incubated with DCFH-DA at 37 °C for 20 min and then detected by FACS analysis, excitation at 488 nm and emission at 535 nm [27].

### Detection of cell energy metabolism

#### Determination of the NAD^+^/NADH ratio

Changes in the intracellular NADH and NAD^+^ concentrations were determined with an NAD^+^/NADH assay kit. Briefly, after 2 h of anthocyanin treatment in 6-well plate, 200 μL of NAD^+^/NADH extract buffer was added into washed cells. After 3 min, cells were collected by centrifugation (12,000 g, 10 min), and then, 100 μL of supernatant was incubated at 60 °C for 30 min to degrade the NAD^+^. With the same operation, 20 μL of incubated sample was transferred into a 96-well plate for NADH detection. Then, 90 μL of ethanol dehydrogenase solution was added to every well and incubated in the dark (37 °C, 10 min) to convert NAD^+^ to NADH. Finally, the samples were incubated in the dark (37 °C, 30 min) with addition of 10 μL of colour reagent before absorbance measurement at 450 nm.

#### ATP levels in cells

An ATP bioluminescence assay kit was used to determine ATP levels in the cells. Briefly, after 2 h of treatment with anthocyanin in 6-well plate, 200 μL lysis buffer was added into the washed cells, followed by centrifugation (10,000 g, 2 min) at 4 °C. Finally, 50 μL of luciferase reagent was mixed with the 50 μL of the supernatant to detect ATP level by a microplate illuminometer (Thermo, USA).

#### Glucose uptake by MC38 cells

Glucose uptake by MC38 cells was determined by a GOD-POD kit. The MC38 cells were seeded into a 24-well plate, and 800 μL of DMEM was added to each well to give a concentration of 5 × 10^5^ cells/well at 37 °C for 24 h. Then medium was removed and washed twice with PBS. After that, 400 μL of sugar-free culture medium was added to each well at 37 °C for 2 h to get starved cells. Then, the sugar-free culture medium was removed and washed twice with PBS. Next, fresh DMEM containing four doses (100, 200, 500 and 1000 μM) of ANC of bilberry extract or anthocyanin standards (cyanidin-3-O-glucoside and cyanidin chloride) was added. After 4 h of ANC treatment, 5 μL of culture medium was added into ELISA plate to measure glucose uptake. Another 5 μL of double distilled water without glucose and 5 μL of standard working fluid was added as blank and standard, respectively. Finally, the samples were incubated in the dark (37 °C, 20 min) with addition of 195 μL of prepared working solution before absorbance measurement at 550 nm.

### Statistical analysis

All data are displayed as mean ± S.D. from triplicate experiments. Significant differences between groups were analysed using one-way analysis of variance (ANOVA) with SPSS 25.0 (Chicago, USA). ¨*¨ indicates statistical significances at *p* < 0.05; ¨**¨, *p* < 0.01; ¨***¨, *p* < 0.001.

## Results and discussion

### Inhibitory effects of the bilberry anthocyanins extract (BAE)

To ensure the same molar levels of anthocyanin were assessed when comparing different groups, we used the term μM to describe the concentration of anthocyanins. As cyanidin-3-glucoside (Cy-3-Glu) is one of the most common anthocyanin enriched in natural plants [28], Cy-3-Glu was used as a standard to determine the concentration of anthocyanin (ANC) of the BAE [29]. The total ANC concentration was calculated as Cy-3-Glu equivalents per L as described in Eqs.:

$$ \mathrm{The}\ \mathrm{concentration}\ \mathrm{of}\ \mathrm{ANC}\ \left(\upmu \mathrm{mol}/\mathrm{L}\right)=\mathrm{M}\ast \mathrm{C}/\mathrm{MW}/\mathrm{V}\ast 1000 $$

where M = quality of added BAE (g); C = 0.36, mass concentration of ANC; MW = 449.2 g/mol for Cy-3-Glu; V = volume of the medium. Final results were expressed as μM Cy-3-Glu equivalents. The effect of BAE was evaluated on MC38 tumour cells and normal cells. As L929 cells are frequently used as normal control cells to detect apoptotic effects [30–32], we used L929 cells as the model of normal cells. According to the data in the literature [2, 33, 34] and the experimental results of IC50 (Table S2), four doses (100, 200, 500 and 1000 μM) of ANC were used for the MTT assays. The oncology drug 5-FU (50 μg/mL) was used as a positive control in all experimental designs.

**Fig. 1:**
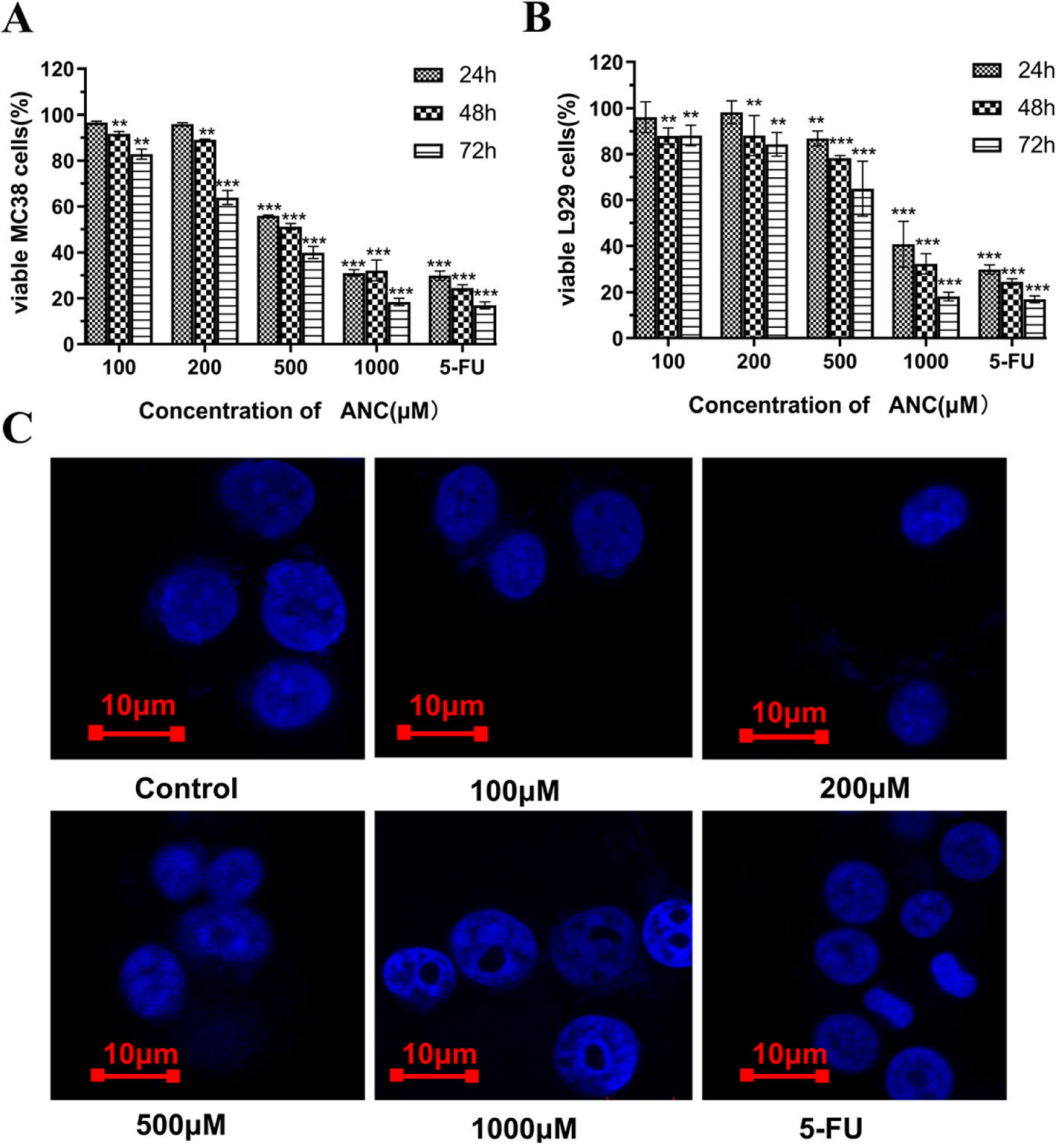
The inhibitory effect of BAE and positive control after 24 h, 48 h, and 72 h of treatment on MC38 cells (**a**) and L929 cells (**b**). Laser confocal microscopy images of Hoechst 33258 in MC38 cells (**c**). Data are displayed as the mean ± S.D. of three independent experiments. *p < 0.05, ** *p* < 0.01, *** *p* < 0.001 respect to control cells by ANOVA

Figure 1a shows that the inhibitory effect of BAE on MC38 cells is dose- and time-dependent, and a significant inhibitory effect appeared when the ANC concentration was 500 μM. The 24 h ANC treatment decreased the MC38 cell viability from 500 to 1000 μM, showing a mean reduction of approximately 55.97 ± 0.26% and 31.09 ± 1.47%, respectively. The extension of treatment time resulted in a more significant effect, particularly with low concentrations of ANC (100 μM and 200 μM). Moreover, high selectivity of ANC towards MC38 cells (survival rate: 55.97 ± 0.26%, 24 h) and L929 cells (survival rate: 86.84 ± 3.26%, 24 h) was achieved at a dose of 500 μM. As the concentration goes up to 1000 μM, ANC showed lower selectivity of inhibitory effect on the growth of MC38 cells (survival rate: 29.95 ± 1.39%, 24 h) and L929 cells (survival rate: 40.84 ± 4.26%, 24 h). Cell apoptosis was determined by observing the apoptotic bodies with laser confocal microscopy after staining with Hoechst 33258, in which normal cell nuclei appeared pale blue and round in shape. When the ANC doses were increased to 500 μM and 1000 μM, apoptotic bodies were observed after 24 h of treatment (Fig. 1e). The incomplete nucleus and blue fluorescent fragments indicated that apoptosis of colonic cancer cells occurred. These results revealed that the BAE induced cell apoptosis.

**Fig. 2:**
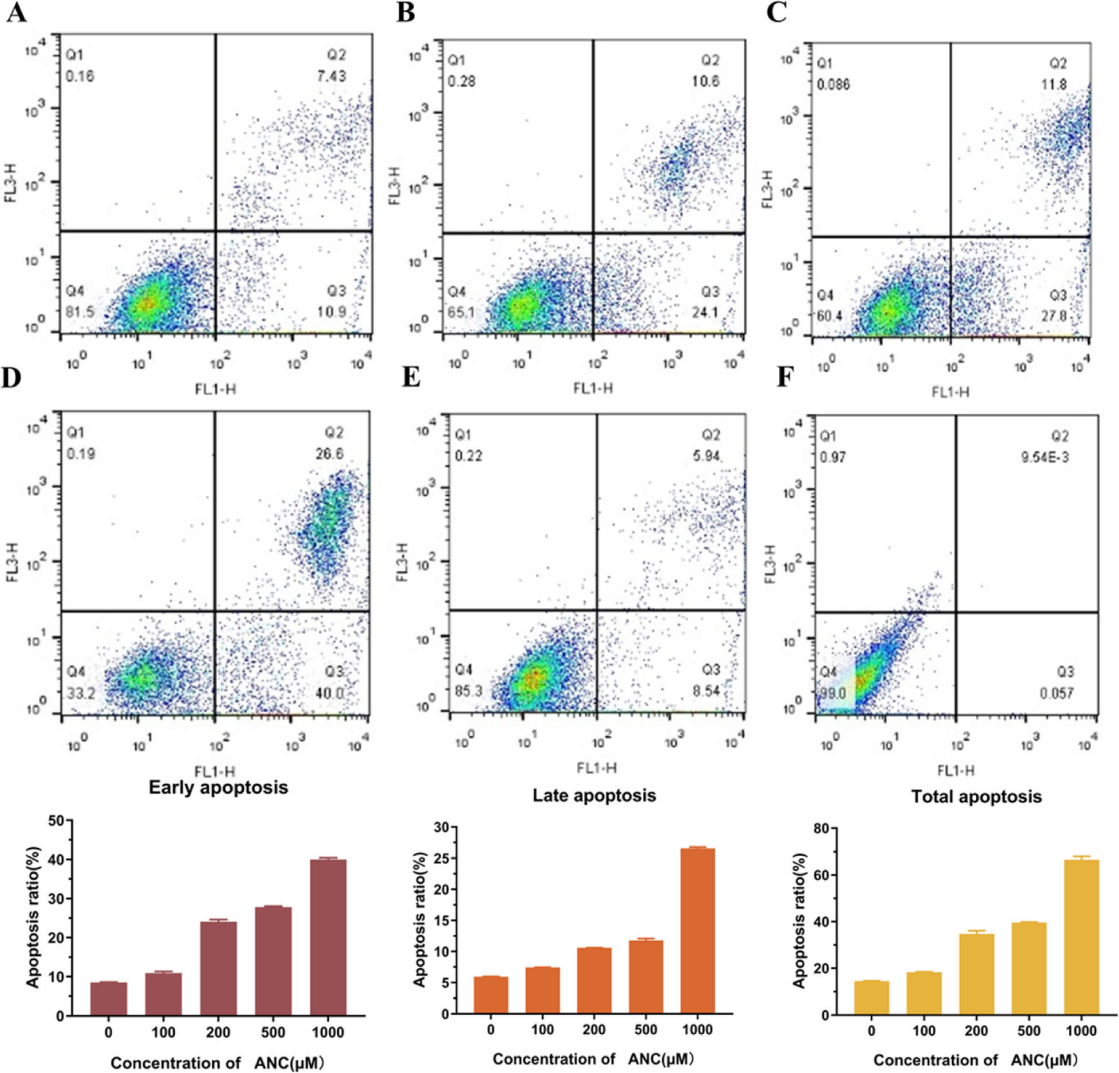
MC38 cells were treated with BAE at different ANC concentrations: (**a**) 100 μM; (**b**) 200 μM; (**c**) 500 μM; (**d**) and 1000 μM; (**e**) and the control and (**f**) negative control for 48 h. The fluorescence intensity ratio was used to show the degree of apoptosis measured by FACS analysis. Data are displayed as the mean ± S.D. of three independent experiments. *p < 0.05, ** *p* < 0.01, *** *p* < 0.001 respect to control cells by ANOVA

The cell apoptosis ratio (Fig. 2, Fig. S2) showed that the BAE could induce cell apoptosis. The population of cells in Q3 and Q2 denotes the percentage of cell apoptosis in the early and late stages, respectively. The percentage of apoptotic cells was significantly increased with the BAE treatment compared to the control (untreated: 14.5 ± 0.14%) after 48 h of incubation (Fig. 2). When the ANC concentration was below 100 μM, the cells showed normal growth. As the content of ANC rose to 200 μM, 24.1 ± 0.57% of the cells entered early apoptosis, and 10.6% of the cells entered late apoptosis. When the ANC concentration was increased to 500 μM, the ratio of early apoptotic cells grew to 27.8 ± 0.28%, but the ratio of late apoptotic cells did not increase obviously. Although the inhibitory effect of low concentrations of ANC was not significant, these treatments led to an increasement in the proportion of cells in early apoptosis. This result was in accordance with the MMP decrease, as shown in previous experiments. When the concentration of ANC was 1000 μM, the proportion of cells in late apoptosis was 40.0 ± 0.42%, and only 33.2 ± 0.05% of the cells still showed normal growth.

### ANCs induce cell apoptosis through mitochondrial depolarisation

#### Mitochondrial depolarisation

Mitochondria are responsible for supporting life under aerobic conditions and are thus considered the source of signals initiating apoptotic cell death. During apoptosis, the decrease in MMP serves as a landmark event in early apoptosis [35]. To further explore the mechanism of apoptosis, we detected decreases in MMP by FACS. When the cell membrane potential is reduced, the transformation of JC-1 is easily detected by the change from red fluorescence to green fluorescence. Thus, the ratio of mitochondrial depolarisation was determined by the relative ratio of red-green fluorescence.

**Fig. 3:**
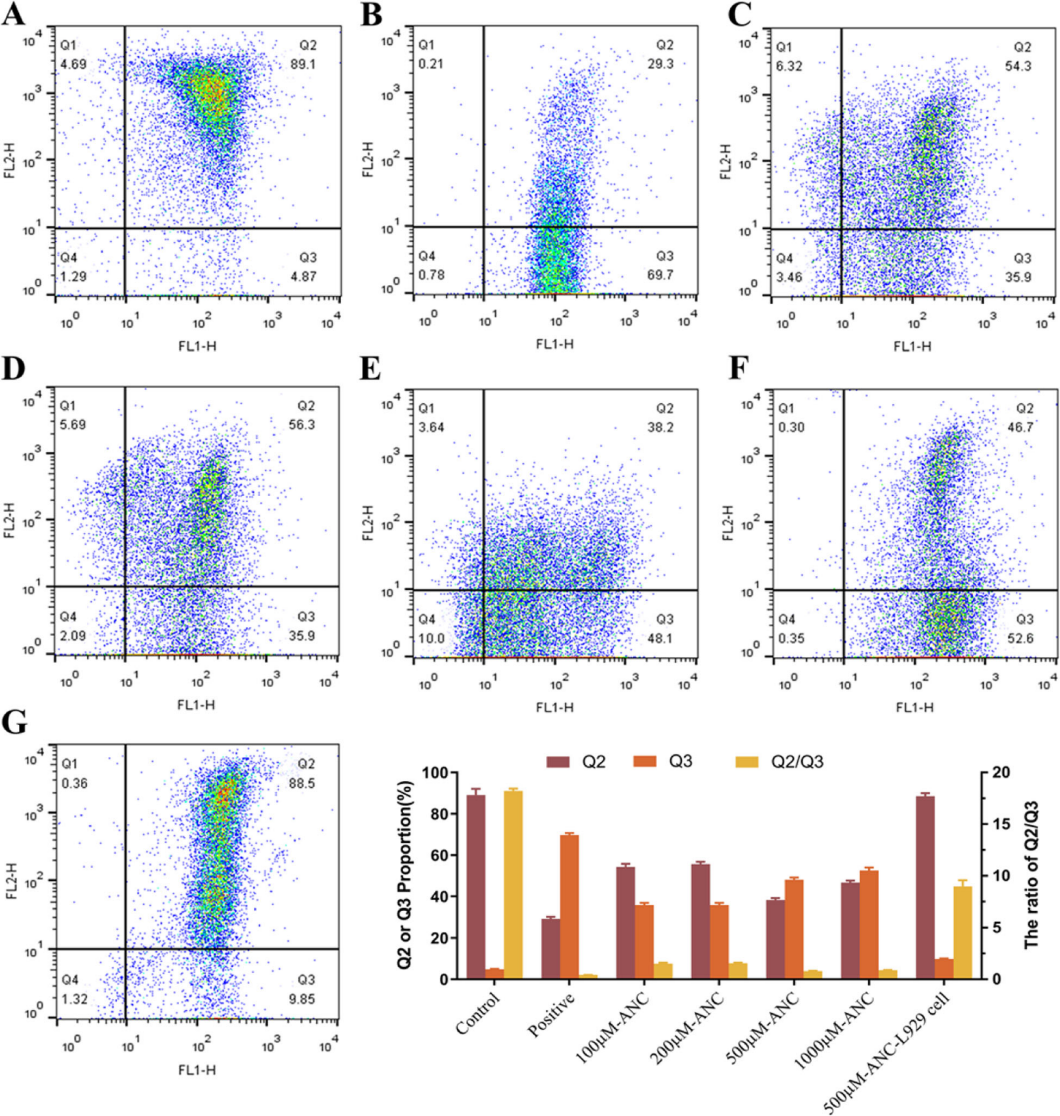
Mitochondrial depolarisation of MC38 cells stained with JC-1 is shown by an increased ratio of the green/red fluorescence intensity measured by FACS analysis after 24 h-treatment with BAE at different ANC concentrations: (**a**) control; (**b**) positive; (**c**) 100 μM ANC; (**d**) 200 μM ANC; (**e**) 500 μM ANC; (**f**) 1000 μM ANC; (**g**) 500 μM BAE-L929 cells. Data are displayed as the mean ± S.D. of three independent experiments. *p < 0.05, ** *p* < 0.01, *** *p* < 0.001 respect to control cells by ANOVA

The mitochondrial membrane potential is relatively higher in control group of MC38 cells stained with JC-1 (Fig. 3a), while the MMP of the positive control group dropped rapidly (Fig. 3b). The aggregated JC-1 (red fluorescence) within normal mitochondria was dissociated into monomeric form (shown with green fluorescence) after treatment with BAE of different ANC concentrations for 24 h, indicating a decrease in MMP and mitochondrial damage. The decrease in MMP was correlated with the increase in ANC concentration. Low concentrations of ANC (e.g.*,* 100 μM, Fig. 3c) resulted in a distinguishable ratio of cells (35.9 ± 1.00%) with low mitochondrial membrane potential, although it had little effect on cell viability.

According to the results shown in Fig. 1, ANC shows selectivity between tumour cells and normal cells at concentrations below 500 μM. Here, we investigated the mitochondrial membrane potential of L929 cells at an ANC concentration of 500 μM. As shown in Fig. 3g, only 9.9 ± 0.20% of the cells had a low membrane potential, which was consistent with the survival rate. The selectivity between tumour cells and normal cells might be attributed to the fact that the energy metabolism of tumour cells is more vigorous than that of normal cells.

#### Activation of caspases

The reduction in mitochondrial membrane potential after apoptosis induces changes in membrane permeability. With the increase in membrane permeability, some apoptosis-inducing factors including cytochrome c are released from the mitochondrial matrix into the cytoplasm. There are key regulators of caspases in mitochondria, which are major factors in many apoptotic processes. The leakage of cytochrome c indicates the disassembly of the apoptosome, which is based on the activation of downstream caspases [36]. As activation of the caspase cascade could lead to a series of events during cell apoptosis, it plays a crucial role in a variety of apoptotic pathways. The caspase protease family consist of initiative group and executive group during apoptotic process [37]. The initiation of mitochondria-mediated apoptotic pathway by caspase-9 resulting in executing apoptosis by caspase-3 [38]. Hence, we detected the caspase-3 and caspase-9 activity using Caspase Activity Assay Kits to further explore the mechanism of apoptosis [39, 40].

**Fig. 4:**
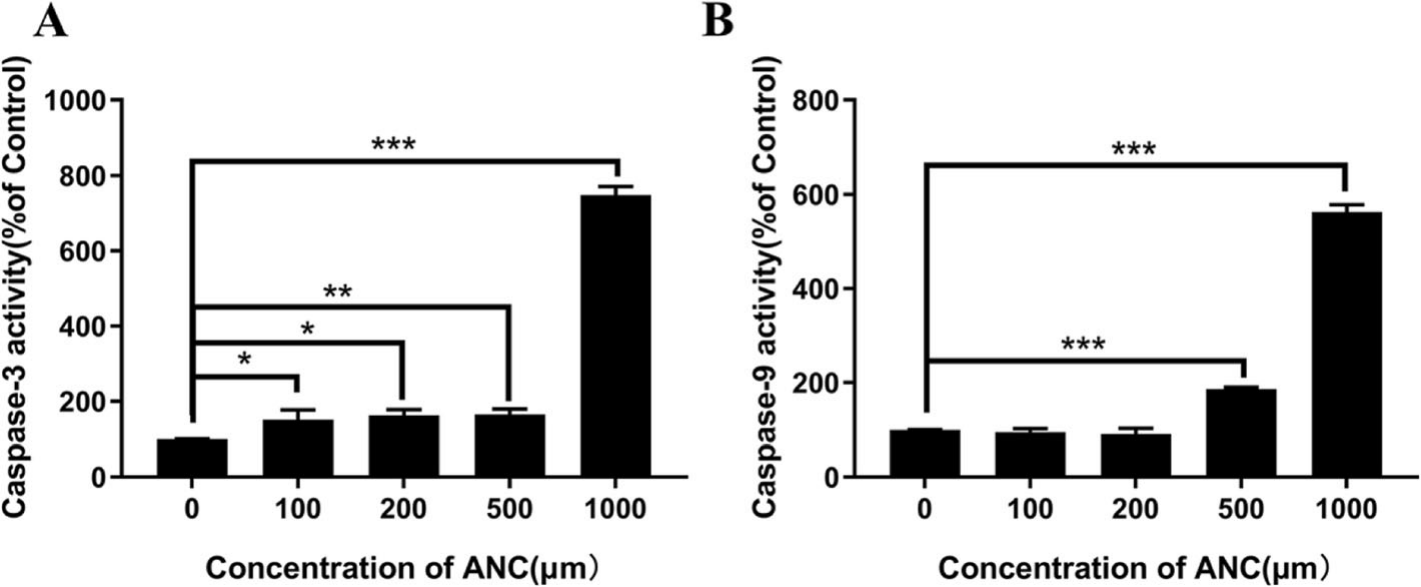
Intracellular enzyme activity changes of MC38 in caspase-3 (A) and caspase-9 (B) after 48 h-treatment with BAE. Data are displayed as the mean ± S.D. of three independent experiments. *p < 0.05, ** *p* < 0.01, *** *p* < 0.001 respect to control cells by ANOVA

Figure 4a reveals the changes of caspase-3 activity in MC38 cells after exposing to BAE with different ANC concentrations for 48 h. The activity of caspase-3 increased to 132.5 ± 2.3%, 155.1 ± 3.6%, 169.4 ± 2.3% and 764.5 ± 3.0% for ANC doses of 100, 200, 500, and 1000 μM compared with the control, respectively. Irreversible morphological changes of cells occurred when the activity of caspase-3 accumulated to a certain threshold. In addition, caspase-3 is the junction between the mitochondrial pathway and the death receptor pathway [41]. This fact explains why the activity of caspase-3 changes in accordance with early cell apoptosis (Fig. 2). Figure 4b presents the changes of caspase-9 activity after 48 h of treatment with ANC concentrations of 100, 200, 500, and 1000 μM. The caspase-9 activity had negligible changes when the ANC concentration was below 500 μM, but a significant increase was found when the ANC concentration was 500 μM (186.0 ± 4.7%). As caspase-9 is an important initiator of apoptosis, its enzyme activity coincides with late apoptosis (Fig. 2). The changes in caspase-3/9 activity correlate with early cell apoptosis (Fig. 1a, Fig. 2), indicating that cell apoptosis proceeds through the mitochondrial route.

### Mitochondrial damage arising from inhibited energy metabolism

**Fig. 5:**
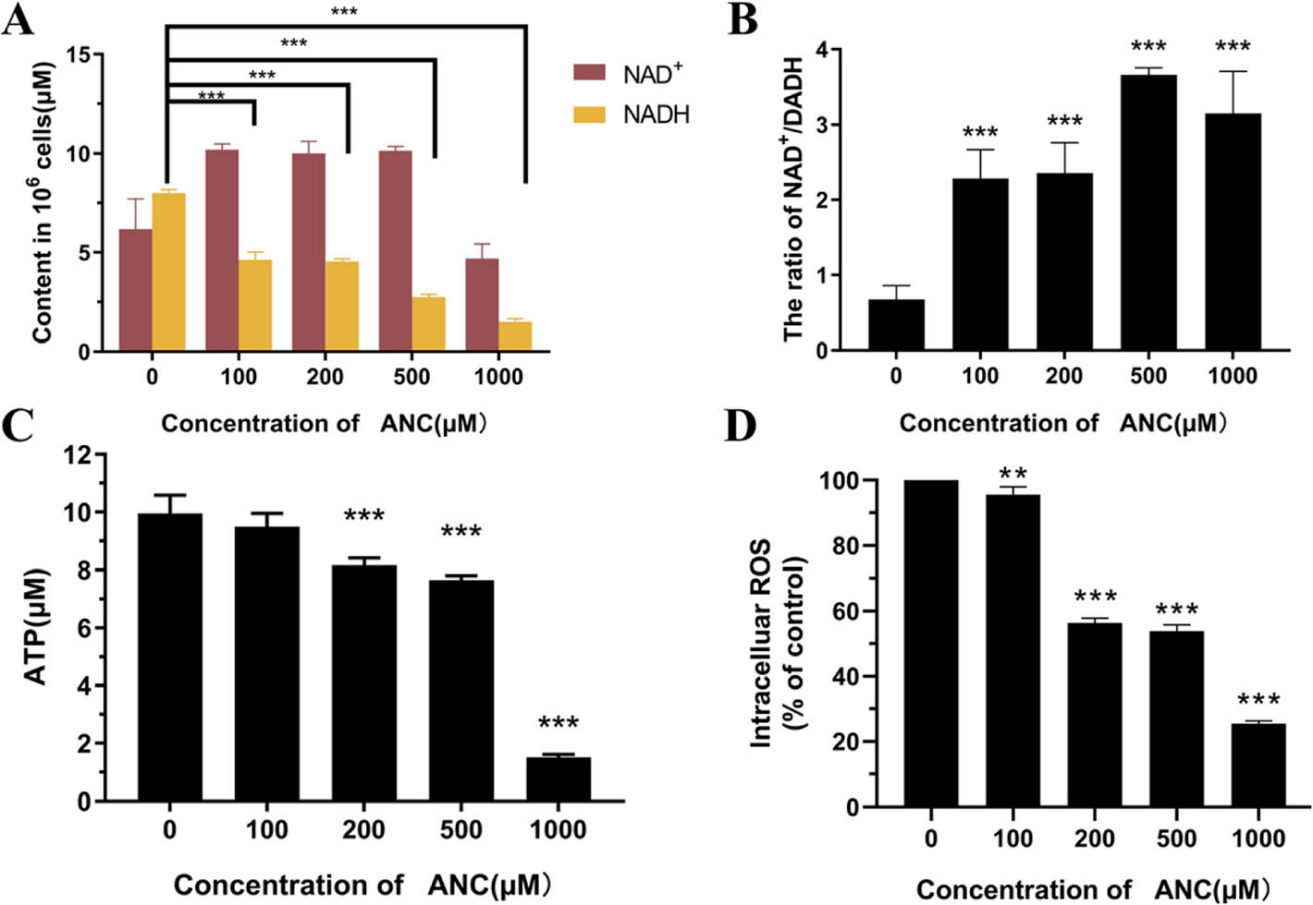
Contents of NADH (**a**), ATP (**c**), and intracellular ROS (**d**) and the ratio of NAD+/NADH (**b**) in MC38 cells after exposing to BAE at different ANC contents for 2 h. Data are displayed as the mean ± S.D. of three independent experiments. *p < 0.05, ** *p* < 0.01, *** *p* < 0.001 respect to control cells by ANOVA

The mitochondrial damage often occurs simultaneously with an increase in excessive reactive oxygen species (ROS) [42] on account of blockade of electron transport in the oxidation respiratory chain. Here, we detected the content of intracellular ROS. Figure 5d showed the ROS level in MC38 cells decreased with increasing ANC concentration, suggesting the strong antioxidant capacity of ANC. This result suggests that electron transport in the oxidation respiratory chain was not blocked and mitochondrial damage was not caused by the increase in ROS.

The lack of oxidative phosphorylation substrates could also cause mitochondrial damage. To further explore the effects of ANC on mitochondrial, we assessed indicators of mitochondrial activity, such as the levels of cellular NADH. NAD (nicotinamide adenine dinucleotide) is a coenzyme that exists in all cells. It includes NAD^+^ (the oxidised form) and NADH (the reduced form). NADH is obtained by reduction of the glycolate dinucleotide, which is produced in the cycle of glycofermentation and cellular respiration. This molecule is also a marker in the mitochondrial energy production chain. NADH produced in the mitochondria can be directly used for ATP synthesis. After treatment with ANC for 2 h, changes in the NADH and ATP contents in the cells were detected (Fig. 5). After the 2 h treatment, there was little difference in the cell number; thus, the discrepancy in energy metabolism was caused only by the concentration of ANC. The results showed significant declines in NADH after treatment with ANC (Fig. 5a). When the concentration of ANC increased to 1000 μM, NADH was only 18.6 ± 0.1% of that in normal cells. The ratio of NAD^+^/NADH increased with increasing concentrations of ANC (Fig. 5b). NAD^+^ is mainly distributed in the cytoplasm, while NADH is mainly located in mitochondria [43]. Thus, the concentration of NAD^+^ changed little, but the reduction of NADH would lead to a higher ratio of NAD^+^/NADH. The lower ratio of NAD^+^/NADH could provide the driving force for the oxidation respiratory chain. Therefore, the decrease in this ratio may inhibit cell proliferation.

The ATP content in the cells was determined as a function of ANC loading. As shown in Fig. 5c, when the ANC concentration was below 500 μM, few changes occurred in ATP, which remained 76.7 ± 0.2% of that of the control group after treatment for 2 h at 500 μM. When the ANC concentration was up to 1000 μM, a sharp decline in ATP was observed, with 16.5 ± 0.1% of the control group. These findings provide direct evidence that ANC could damage mitochondria by reducing oxidative phosphorylation agents.

### How glycosides enhance inhibitory activities

**Fig. 6:**
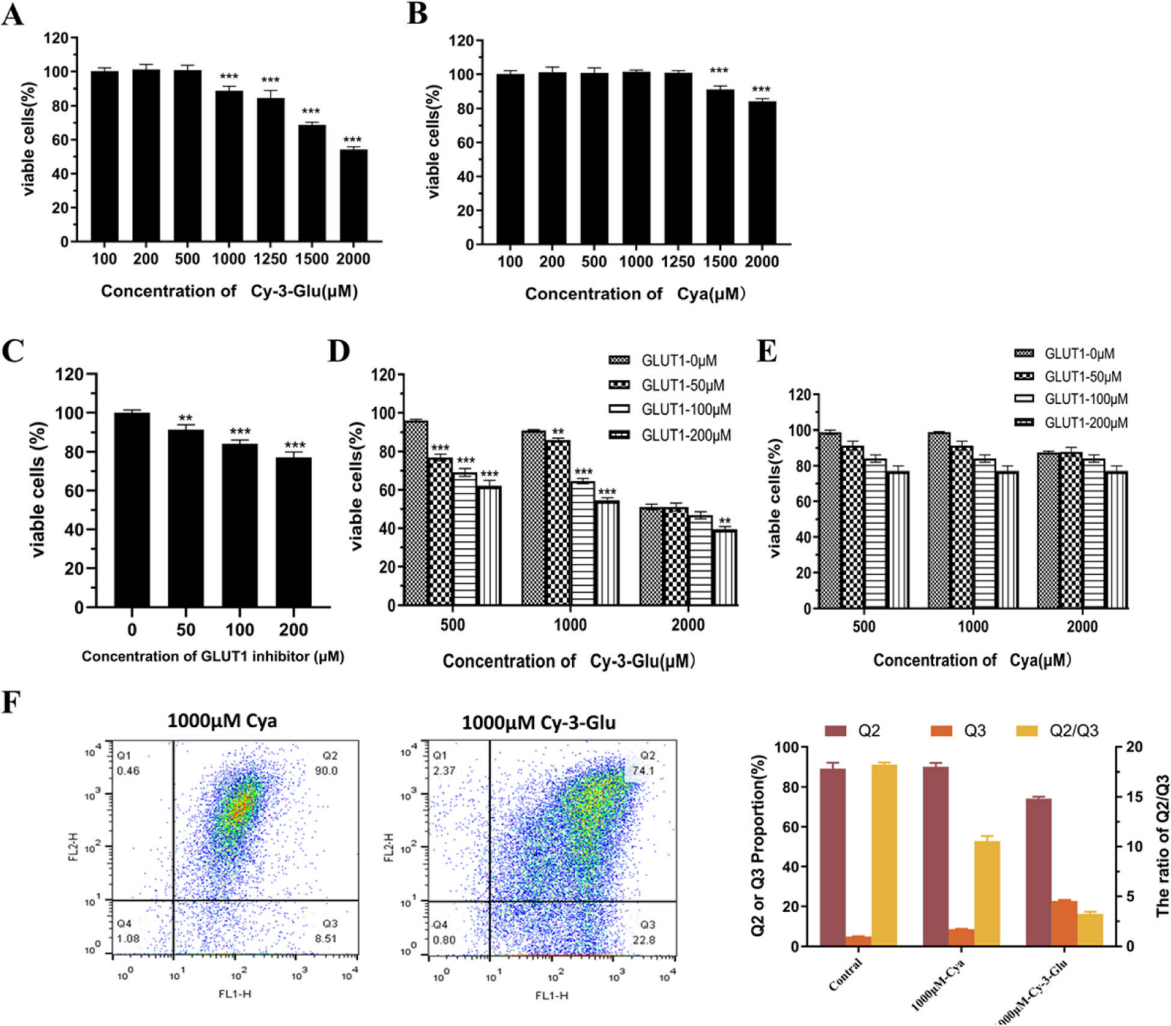
The inhibitory effect of Cy-3-Glu (**a**), Cya (**b**), GLUT1 inhibitor (**c**), the combination of Cy-3-Glu and GLUT1 inhibitor (**d**) and the combination of Cya and GLUT1 inhibitor (**e**) after 24 h-treatment on MC38 cells. Mitochondrial depolarisation is shown by FACS analysis after the MC38 cells were treated with Cy-3-Glu and Cya for 24 h at 1000 μM and cells were stained with JC-1 (**f**). Data are displayed as the mean ± S.D. of three independent experiments. *p < 0.05, ** *p* < 0.01, *** *p* < 0.001 respect to control cells by ANOVA

To identify the impact of glucosides of ANC on the inhibition of MC38 cell proliferation, we chose cyanidin-3-O-glucoside (Cy-3-Glu) and its anthocyanidin aglycone, cyanidin (Cya), as the model anthocyanin and anthocyanidin. Cell proliferation was inhibited when the concentration of cyanidin-3-O-glucoside reached 1000 μM (Fig. 6a). Compared with the results shown in Fig. 1a, ANC showed a better inhibitory effect on Cy-3-Glu at the same concentration, indicating that there might be a synergistic effect among anthocyanin extracts. Similar results have been reported for the mixtures of ANC-rich berry extracts in inhibiting breast cancer cells [44].

As for Cy-3-Glu and Cya, as shown in Fig. 6b, at concentrations reaching 2000 μM, only approximately half of the cells survived after treatment with Cy-3-Glu, while the survival rate was over 80% after treatment with Cya. Here, the existence of glucosides led to a dramatic difference in the inhibitory effect. We then examined the effects of Cy-3-Glu and Cya on mitochondrial membrane potential. Figure 6f shows that only 8.5 ± 0.2% of the MC38 cells treated with 1000 μM Cya for 24 h had damaged mitochondria, whereas Cy-3-Glu led to a nearly 3-fold increase in mitochondrial damage at the same concentration.

Cell energy metabolism is dependent on glucose uptake. These data suggested that the glycosides of anthocyanin might regulate energy metabolism by disturbing glucose transport. It has been demonstrated that anthocyanins extract could regulate intestinal sugar absorption [45], decrease glucose uptake [46, 47] and inhibit cell proliferation due to interference of glucose uptake [48]. Further, a recent study shows that anthocyanin could not only inhibit glucose absorption but also affect the function of sodium-glucose cotransporter 1 (SGLT1) [49]. Johnston K et al. investigated the effect of dietary polyphenols on intestinal glucose uptake and found that glucose uptake into cells under sodium-dependent conditions was inhibited by flavonoid glycosides whereas aglycones and phenolic acids were without effect [50]. These all points out the importance of glycosylation on glucose uptake.

**Fig. 7:**
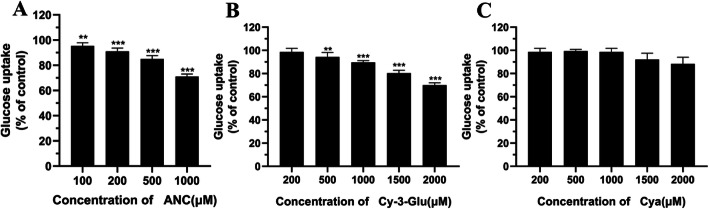
Glucose uptake in MC38 cells in the presence of BAE (**a**), Cy-3-Glu (**b**) or Cya (C) after exposing to anthocyanin at different contents for 4 h. Data are displayed as the mean ± S.D. of three independent experiments. *p < 0.05, ** *p* < 0.01, *** *p* < 0.001 respect to control cells by ANOVA

Our experimental results using MC38 cells also demonstrated that the glucose transport to MC38 cells was hadicapped by anthocyanin. As shown in Fig. 7, ANC and Cy-3-Glu caused a significant reduction in glucose uptake and in a dose-dependent manner. Compare the cell survival test, the glucose transport efficiency is consistent with the cell survival rate. However, the model aglycones (Cya) only had slighter inhibitory effect on glucose uptake at a high concentration level (93.3% reduction at 1500 μM). These observations suggest glycosylation of anthocyanin significantly enhanced the inhibitory effect of glucose uptake. A potential mechanism might be that the glycosides of anthocyanin affect the glucose transporter GLUT1, which is reported to regulate the glucose uptake of tumour cells and maintain basal metabolism [51, 52].

Furthermore, we added the WZB117 (GLUT1 inhibitor) to culture medium containing Cy-3-Glu or Cya at various doses and determined the cell viability. As an GLUT1 inhibitor, WZB117 could downregulate glycolysis, induce cell-cycle arrest [53, 54], and inhibit cancer cell growth in a dose-dependent manner (Fig. 6c). The results (Fig. 6d) showed that the presence of the GLUT1 inhibitor substantially enhanced the inhibitory effects at low concentrations of Cy-3-Glu. Such an enhancement is marginal at higher concentrations of Cy-3-Glu, which suggests saturated binding of GLUT1. Whereas, the addition of the GLUT1 inhibitor couldn’t enhance the inhibitory effects of Cya (Fig. 6e).

**Scheme 1:**
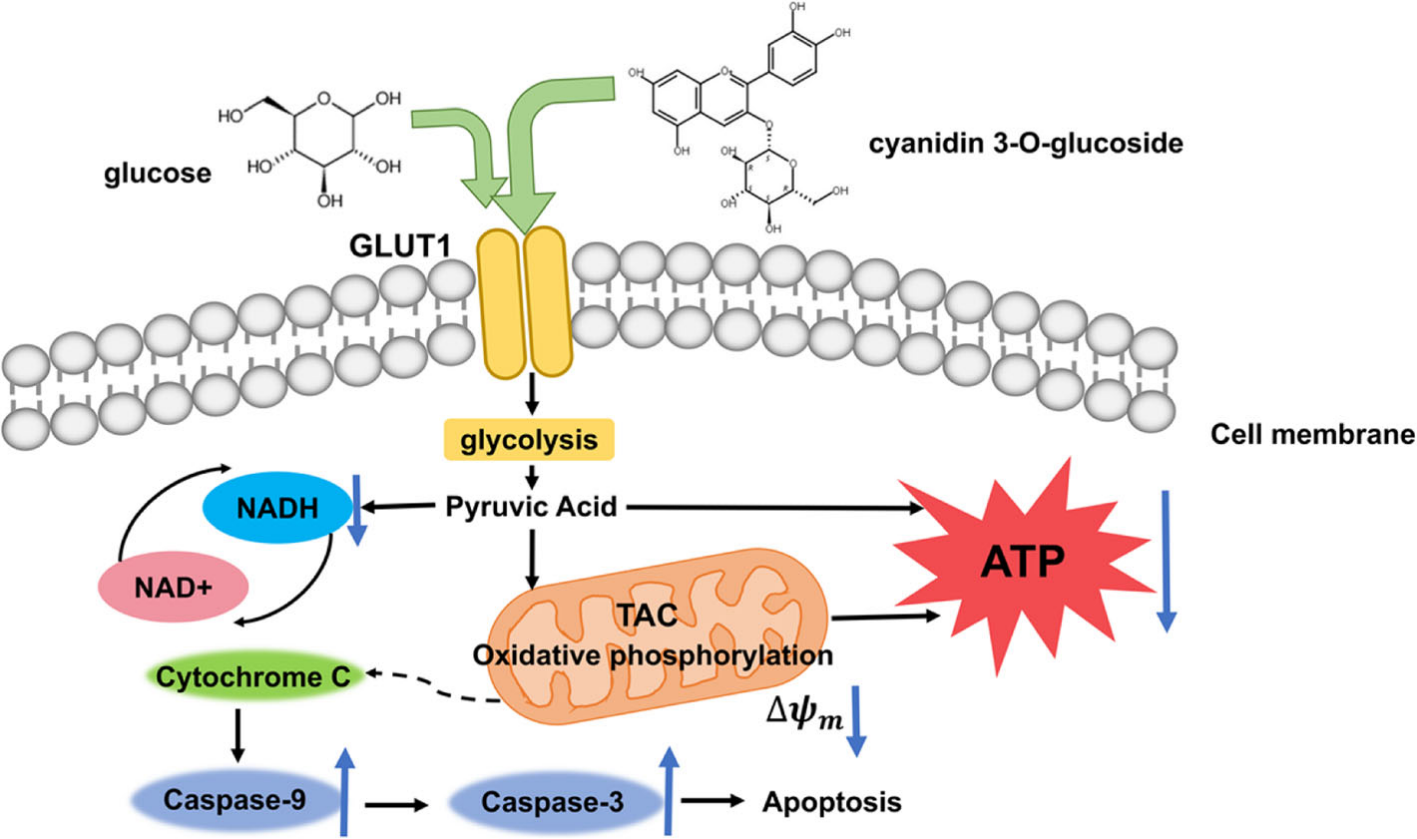
Molecular mechanism of anthocyanin inhibiting cell proliferation

With the above findings, we proposed a mechanism describing the role of glycosides of anthocyanins in inhibiting MC38 cells, as shown in Scheme 1. The glycosides of anthocyanin might affect GLUT1 on the tumour cell membrane and thus hinder glucose uptake by MC38 cells. Consequently, this phenomenon blocks the energy metabolism of tumour cells and leads to mitochondrial damage and subsequent activation of downstream caspases. These effects, together with the function of the anthocyanidin aglycone, which impacts several signalling pathways in cell growth and function, initiate the apoptosis of tumour cells. Since tumour cells have a more vigorous energy metabolism than normal cells, the obstruction of energy metabolism produces more serious impacts on cell growth of tumour cells, which contributes to the selective inhibition between tumour cells and normal cells.

## Conclusions

Anthocyanins inhibited MC38 cells through a process from early apoptosis to late apoptosis, but the anthocyanidin aglycone had little inhibitory effect on MC38 cells. An experimental study showed that anthocyanins induced the loss of MMP, indicating mitochondrial damage, and activated the downstream caspase cascade to form apoptotic bodies. However, anthocyanidin could barely induce mitochondrial damage. Further studies demonstrated that the inducer of mitochondrial damage was not the ROS but the inhibition of energy metabolism, as evidenced by the increased ratio of NAD^+^/NADH and the decreased ATP level. The comparative study using Cy-3-Glu and Cya suggested that the glycosides of anthocyanin might inhibit cell energy metabolism of the cell and thus induce cell apoptosis. Based on the aforementioned findings, we propose that the inhibitory effect of anthocyanin on tumour cell proliferation might be contributed jointly by anthocyanidin aglycone, which affects several signalling pathways, and anthocyanin glycosides, which inhibit energy metabolism and lead to mitochondrial damage. These findings are essential for the design, processing and application of anthocyanin products with healthy attributes or medical functions.

## Supplementary information

**Additional file 1.**

## Data Availability

All data contained within the manuscript as well as supporting materials are available from the corresponding author on reasonable request.

## Abbreviations

ANC: Anthocyanin
BAE: bilberry anthocyanins extract
Cy-3-glu: cyanidin-3-O-glucoside
Cya: cyanidin
GLUT: glucose transporters
SGLT1: glucose co-transporter
MMP: mitochondrial membrane potential
MMPs: metalloproteinases
DMEM: Dulbecco’s modified Eagle’s medium
FACS: flow cytometry
DCFH-DA: 2′, 7′-dichlorofluorescein diacetate
DCF: fluorescent dichlorofluorescein
ROS: reactive oxygen species
NAD: nicotinamide adenine dinucleotide
NAD^+^: the oxidised form
MTT: 3-(4,5-dimthyl-2-thiazolyl)-2,5-diphenyl-2-H-tetrazolium bromide
DMSO: dimethyl sulfoxide
5-FU: 5-fluorouracil

## References

[1] McGhie TK, Walton MC (2007). The bioavailability and absorption of anthocyanins: towards a better understanding. Mol Nutr Food Res.

[2] Nguyen V, Tang J, Oroudjev E, Lee CJ, Marasigan C, Wilson L, Ayoub G (2010). Cytotoxic effects of bilberry extract on MCF7-GFP-tubulin breast Cancer cells. J Med Food.

[3] Li J, Wu T, Li N, Wang XN, Chen GY, Lyu XL (2019). Bilberry anthocyanin extract promotes intestinal barrier function and inhibits digestive enzyme activity by regulating the gut microbiota in aging rats. Food Funct.

[4] Olejnik A, Kowalska K, Kidon M, Czapski J, Rychlik J, Olkowicz M, Dembczynski R (2016). Purple carrot anthocyanins suppress lipopolysaccharide-induced inflammation in the co-culture of intestinal Caco-2 and macrophage RAW264.7 cells. Food Funct.

[5] Venancio VP, Cipriano PA, Kim H, Antunes LMG, Talcott ST, Mertens-Talcott SU (2017). Cocoplum (Chrysobalanus icaco L.) anthocyanins exert anti-inflammatory activity in human colon cancer and non-malignant colon cells. Food Funct.

[6] Seeram NP (2008). Berry fruits: compositional elements, biochemical activities, and the impact of their intake on human health, performance, and disease. J Agric Food Chem.

[7] Zafra-Stone S, Yasmin T, Bagchi M, Chatterjee A, Vinson JA, Bagchi D (2007). Berry anthocyanins as novel antioxidants in human health and disease prevention. Mol Nutr Food Res.

[8] Cooke D, Steward WP, Gescher AJ, Marczylo T (2005). Anthocyans from fruits and vegetables - does bright colour signal cancer chemopreventive activity?. Eur J Cancer.

[9] Pan FG, Liu YJ, Liu JB, Wang EL (2019). Stability of blueberry anthocyanin, anthocyanidin and pyranoanthocyanidin pigments and their inhibitory effects and mechanisms in human cervical cancer HeLa cells. RSC Adv.

[10] Li DT, Wang PP, Luo YH, Zhao MY, Chen F (2017). Health benefits of anthocyanins and molecular mechanisms: update from recent decade. Crit Rev Food Sci Nutr.

[11] Li H, Fan Y, Zhang L, Liu A, Tu F, He K, Zhang J (2016). Phenethyl isothiocyanate inhibits the migration and invasion of colon cancer SW480 cells via the inhibition of matrix metalloproteinase-9. Int J Clin Exp Med.

[12] Li X, Zhang ZS, Zhang XH, Yang SN, Liu D, Diao CR, Wang H, Zheng FP (2019). Cyanidin inhibits EMT induced by oxaliplatin via targeting the PDK1-PI3K/Akt signaling pathway. Food Funct.

[13] Malik M, Zhao CW, Schoene N, Guisti MM, Moyer MP, Magnuson BA (2003). Anthocyanin-rich extract from Aronia meloncarpa E. induces a cell cycle block in colon cancer but not normal colonic cells. Nutr Cancer.

[14] Xiao JB, Muzashvili TS, Georgiev MI (2014). Advances in the biotechnological glycosylation of valuable flavonoids. Biotechnol Adv.

[15] Bai N, He K, Roller M, Lai CS, Shao X, Pan MH, Bily A, Ho CT (2011). Flavonoid glycosides from Microtea debilis and their cytotoxic and anti-inflammatory effects. Fitoterapia..

[16] Yu CH, Zhang ZY, Zhang HJ, Zhen Z, Calway T, Wang YW, Yuan CS, Wang CZ (2013). Pretreatment of baicalin and wogonoside with glycoside hydrolase: a promising approach to enhance anticancer potential. Oncol Rep.

[17] Mamadalieva NZ, Herrmann F, El-Readi MZ, Tahrani A, Hamoud R, Egamberdieva DR, Azimova SS, Wink M (2011). Flavonoids in Scutellaria immaculata and S. ramosissima (Lamiaceae) and their biological activity. J Pharm Pharmacol.

[18] Kamiloglu S, Capanoglu E, Grootaert C, Van Camp J (2015). Anthocyanin absorption and metabolism by human intestinal Caco-2 cells--a review. Int J Mol Sci.

[19] Zou TB, Feng D, Song G, Li HW, Tang HW, Ling WH (2014). The role of sodium-dependent glucose transporter 1 and glucose transporter 2 in the absorption of cyanidin-3-o-beta-glucoside in Caco-2 cells. Nutrients..

[20] Manzano S, Williamson G (2010). Polyphenols and phenolic acids from strawberry and apple decrease glucose uptake and transport by human intestinal Caco-2 cells. Mol Nutr Food Res.

[21] Alzaid F, Cheung H-M, Preedy VR, Sharp PA. Regulation of Glucose Transporter Expression in Human Intestinal Caco-2 Cells following Exposure to an Anthocyanin-Rich Berry Extract. PLoS One. 2013;8(11):1–6. 10.1371/journal.pone.0078932. 10.1371/journal.pone.0078932 PMC3827299 24236070

[22] Cassinese C, De Combarieu E, Falzoni M, Fuzzati N, Pace R, Sardone N (2007). New liquid chromatography method with ultraviolet detection for analysis of anthocyanins and anthocyanidins in Vaccinium myrtillus fruit dry extracts and commercial preparations. J AOAC Int.

[23] Gowd V, Bao T, Wang L, Huang Y, Chen S, Zheng X, Cui S, Chen W (2018). Antioxidant and antidiabetic activity of blackberry after gastrointestinal digestion and human gut microbiota fermentation. Food Chem.

[24] Chen W, Zhang L, Zhang K, Zhou B, Kuo M-L, Hu S, Chen L, Tang M, Chen Y-R, Yang L (2014). Reciprocal regulation of autophagy and dNTP pools in human cancer cells. Autophagy..

[25] Hu D, Xu Y, Xie J, Sun C, Zheng X, Chen W (2018). Systematic evaluation of phenolic compounds and protective capacity of a new mulberry cultivar J33 against palmitic acid-induced lipotoxicity using a simulated digestion method. Food Chem.

[26] Chen W, Feng L, Shen Y, Su H, Li Y, Zhuang J, Zhang L, Zheng X. Myricitrin inhibits acrylamide-mediated cytotoxicity in human Caco-2 cells by preventing oxidative stress. Biomed Res Int. 2013;2013:1–7. 10.1155/2013/724183. PMC3809942 24224177

[27] Wang YF, Fang F, Wong CW (2010). Troglitazone is an estrogen-related receptor a and gamma inverse agonist. Biochem Pharmacol.

[28] Liang LW, Liu XP, He JY, Shao Y, Liu J, Wang ZY, Xia LN, Han T, Wu PY (2019). Cyanidin-3-glucoside induces mesenchymal to epithelial transition via activating Sirt1 expression in triple negative breast cancer cells. Biochimie..

[29] Mazewski C, Liang K, Gonzalez de Mejia E. Inhibitory potential of anthocyanin-rich purple and red corn extracts on human colorectal cancer cell proliferation in vitro. J Funct Foods 2017;34:254–265.

[30] Moskvin M, Babic M, Reis S, Margarida Cruz M, Ferreira LP, Carvalho MD, Costa Lima SA, Horak D (2018). Biological evaluation of surface-modified magnetic nanoparticles as a platform for colon cancer cell theranostics. Colloids and Surfaces B-Biointerfaces.

[31] Hosseini FS, Noroozi Karimabad M, Hajizadeh MR, Khoshdel A, Khanamani Falahati-Pour S, Mirzaei MR, Mirmohamadi SM, Mahmoodi M (2019). Evaluating of Induction of Apoptosis by Cornus mass L. Extract i..n the Gastric Carcinoma Cell Line (AGS). Asian Pac J Cancer Prev.

[32] Rzayev ZMO, Khalilova S, Salimi K (2017). Folic acid conjugated and Ag-carrying organoclay nanoparticles and their response to L929 fibroblast and DLD-1 cancer cells. J Microencapsul.

[33] Amatori S, Mazzoni L, Alvarez-Suarez JM, Giampieri F, Gasparrini M, Forbes-Hernandez TY, Afrin S, Errico Provenzano A, Persico G, Mezzetti B (2016). Polyphenol-rich strawberry extract (PRSE) shows in vitro and in vivo biological activity against invasive breast cancer cells. Sci Rep..

[34] Long HL, Zhang FF, Wang HL, Yang WS, Hou HT, Yu JK, Liu B (2018). Mulberry anthocyanins improves thyroid cancer progression mainly by inducing apoptosis and autophagy cell death. Kaohsiung J Med Sci.

[35] Green DR, Reed JC (1998). Mitochondria and apoptosis. Science..

[36] Cain K, Bratton SB, Cohen GM (2002). The Apaf-1 apoptosome: a large caspase-activating complex. Biochimie..

[37] Zhang F, Shi JJ, Thakur K, Hu F, Zhang JG, Wei ZJ. Anti-cancerous potential of polysaccharide fractions extracted from Peony seed dreg on various human Cancer cell lines via cell cycle arrest and apoptosis. Front Pharmacol. 2017;8(102):1–13. 10.3389/fphar.2017.00102. 10.3389/fphar.2017.00102 PMC5334287 28316571

[38] Lee D-H, Kim D-W, Jung C-H, Lee YJ, Park D (2014). Gingerol sensitizes TRAIL-induced apoptotic cell death of glioblastoma cells. Toxicol Appl Pharmacol.

[39] Li M, Song M, Ren L-M, Xiu C-Y, Liu J-Y, Zhu Y-Z, Li Y-F. AlCl3 induces lymphocyte apoptosis in rats through the mitochondria-caspase dependent pathway. Environ Toxicol 2016;31(4):385–394. 10.1002/tox.22051 25263842

[40] Yu G, Li N, Zhao Y, Wang W, Feng X-L (2018). Salidroside induces apoptosis in human ovarian cancer SKOV3 and A2780 cells through the p53 signaling pathway. Oncol Lett.

[41] Porter AG, Janicke RU (1999). Emerging roles of caspase-3 in apoptosis. Cell Death Differ.

[42] Sanderson TH, Reynolds CA, Kumar R, Przyklenk K, Huttemann M (2013). Molecular mechanisms of ischemia-reperfusion injury in brain: pivotal role of the mitochondrial membrane potential in reactive oxygen species generation. Mol Neurobiol.

[43] Williamson DH, Lund P, Krebs HA. Redox state of free nicotinamide-adenine dinucleotide in cytoplasm and mitochondria of rat liver. Biochem J. 1967;103(2):514. 10.1042/bj1030514 PMC1270436 4291787

[44] Wang S, Zhu F, Marcone MF (2015). Synergistic interaction of sumac and raspberry mixtures in their antioxidant capacities and selective cytotoxicity against cancerous cells. J Med Food.

[45] Barik SK, Russell WR, Moar KM, Cruickshank M, Scobbie L, Duncan G, Hoggard N (2020). The anthocyanins in black currants regulate postprandial hyperglycaemia primarily by inhibiting alpha-glucosidase while other phenolics modulate salivary alpha-amylase, glucose uptake and sugar transporters. J Nutrit Biochem.

[46] Mojica L, Berhow M, de Mejia EG (2017). Black bean anthocyanin-rich extracts as food colorants. Physicochemical stability and antidiabetes potential. Food Chem.

[47] Johnston K, Sharp P, Clifford M, Morgan L (2005). Dietary polyphenols decrease glucose uptake by human intestinal Caco-2 cells. FEBS Lett.

[48] Faria A, Pestana D, Azevedo J, Martel F, de Freitas V, Azevedo I, Mateus N, Calhau C (2009). Absorption of anthocyanins through intestinal epithelial cells - putative involvement of GLUT2. Mol Nutr Food Res.

[49] Hidalgo J, Teuber S, Morera FJ, Ojeda C, Flores CA, Hidalgo MA, Nunez L, Villalobos C, Burgos RA (2017). Delphinidin reduces glucose uptake in mice Jejunal tissue and human intestinal cells lines through FFA1/GPR40. Int J Mol Sci.

[50] Castro-Acosta ML, Hall WL, Corpe CP (2016). Polyphenol-rich blackcurrant and apple extracts inhibit glucose uptake in in vitro models of intestinal sugar transport, but individual anthocyanins have no effect. Proc Nutr Soc..

[51] Jurcovicova J (2014). Glucose transport in brain - effect of inflammation. Endocr Regul.

[52] Kapoor S (2013). Glucose transporter 1 (GLUT1) and its emerging role as a significant prognostic marker in systemic malignancies. Int J Color Dis.

[53] Li Y, Hong W, Zhang H, Zhang TT, Chen Z, Yuan S, Peng P, Xiao M, Xu L (2020). Photothermally triggered cytosolic drug delivery of glucose functionalized polydopamine nanoparticles in response to tumor microenvironment for the GLUT1-targeting chemo-phototherapy. J Control Release.

[54] Liu Y, Cao Y, Zhang W, Bergmeier S, Qian Y, Akbar H, Colvin R, Ding J, Tong L, Wu S (2012). A small-molecule inhibitor of glucose transporter 1 Downregulates glycolysis, induces cell-cycle arrest, and inhibits Cancer cell growth in vitro and in vivo. Mol Cancer Ther.

