# Supplementary material for: Glycosylation of anthocyanins enhances the apoptosis of colon cancer cells by handicapping energy metabolism
Source: BMC Complement Med Ther. 2020 Oct 15;20:312. doi: 10.1186/s12906-020-03096-y (PMC7566133; doi:10.1186/s12906-020-03096-y)
Supplement: Supplementary file 1 — Additional file 1. [file 12906_2020_3096_MOESM1_ESM.docx]

**Supporting information**

1. **HPLC-DAD-MS analysis of anthocyanins extract**

Anthocyanins composition in bilberry extract were analyzed using HPLC-DAD-MS spectrometer (Shimadzu, Japan), using TSK C-18 column of 150 × 4.6 mm kept at 30◦C. Solution A was a mixture of water/formic acid in the ratio of 9:1 (v/v) and solution B was water/methanol/acetonitrile/formic acid in the ratio of 16:9:9:4 (v/v/v/v). 20 μL of sample were injected and HPLC was performed in a gradient mode (0-35min: 93-75%A+7-25%B; 35-45 min: 75-35% A+25-65%B; 45-46 min:35-0% A+65-100% B; 46-50 min: isocratic 93% A + 7% B) with the flow rate of 1 mL/min. PDA detection was set for 200–700 nm. Conditions for mass spectrometry analysis: nitrogen was used as both auxiliary (flow rate: 15 L/min) and sheath (flow rate: 3 L/min) gas; the DL temperature was 250 °C and CID voltage was 230 kPa; Electrospray Ionization Source (ESI) with positive ion scanning. The m/z mass range was 100–1500. The anthocyanins extract was analyzed in triplicate.

**
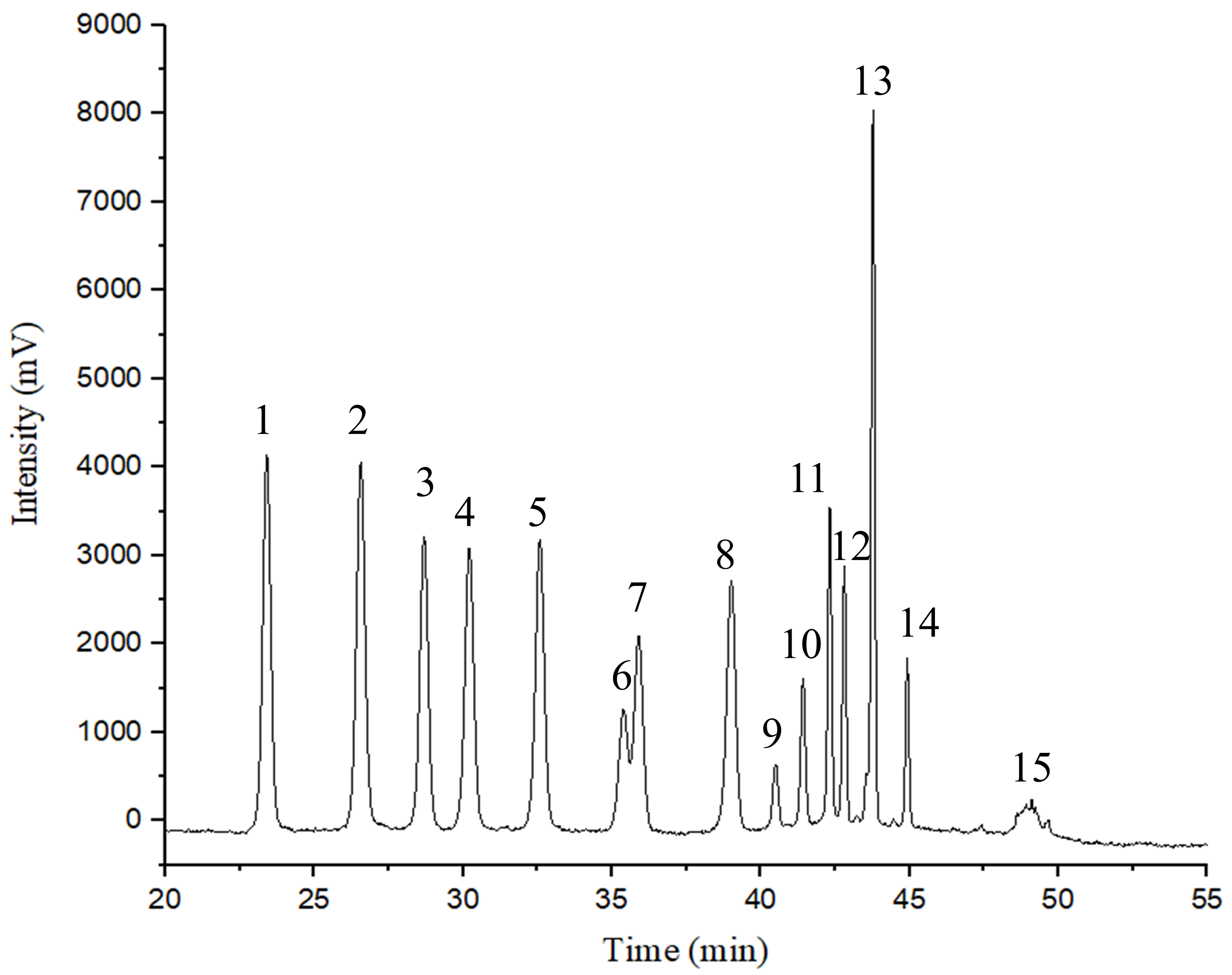
**

**Fig S1.** HPLC profiles of bilberry extract at 535 nm. Peaks assignment:1, Delphindin-3-O-galactoside; 2, Delphindin-3-O-glucoside; 3, Cyanidin-3-O-galactoside; 4, Delphindin-3-O-arabinoside; 5, Cyanidin-3-O-glucoside;6, Petunidin-3-O-galactoside; 7, Cyanidin-3-O-arabinoside; 8, Petunidin-3-O-glucoside; 9, Petunidin-3-O-galactoside; 10, Petunidin-3-O-arabinoside; 11, Peonidin-3-O-glucoside; 12, Malvidin-3-O-glucoside

; 13, Malvidin-3-O-galactoside; 14, Malvidin-3-O-arabinoside; 15, mixture of Petunidin, Peonidin and Malvidin.

**Table S1.** Retention time(R_t_), mass spectral data,total anthocyanins content in bilberry

| Peak | Rt(min) | [M^+^](m/z) | Identification | Percentage (% of total ANC) | |
| --- | --- | --- | --- | --- | --- |
| 1 | 23.166 | 500.84 | Delphinidin-3-O-galactoside | | 11.57±0.12 |
| 2 | 26.286 | 500.84 | Delphinidin-3-O-glucoside | | 12.10±1.31 |
| 3 | 28.392 | 484.84 | Cyanidin-3-O-galactoside | | 9.20±0.14 |
| 4 | 29.900 | 470.81 | Delphindin-3-O-arabinoside | | 9.67±0.44 |
| 5 | 32.232 | 484.84 | Cyanidin-3-O-glucoside | | 9.72±0.07 |
| 6 | 35.001 | 514.88 | Petunidin-3-O-galactoside | | 4.38±0.01 |
| 7 | 35.526 | 454.81 | Cyanidin-3-O-arabinoside | | 11.01±0.56 |
| 8 | 38.601 | 514.86 | Petunidin-3-O-glucoside | | 8.72±0.34 |
| 9 | 40.242 | 498.86 | Petunidin-3-O-galactoside | | 10.25±0.18 |
| 10 | 41.217 | 484.85 | Petunidin-3-O-arabinoside | | 3.00±0.43 |
| 11 | 42.146 | 498.86 | Peonidin-3-O-glucoside | | 5.19±0.22 |
| 12 | 42.465 | 528.89 | Malvidin-3-O-glucoside | | 4.03±0.06 |
| 13 | 43.627 | 528.89 | Malvidin-3-O-galactoside | | 10.91±1.01 |
| 14 | 44.771 | 498.86 | Malvidin-3-O-arabinoside | | 2.53±0.08 |
| 15 | **-** | **-** | Mixture of anthocyanidins | | 0.83±0.02 |


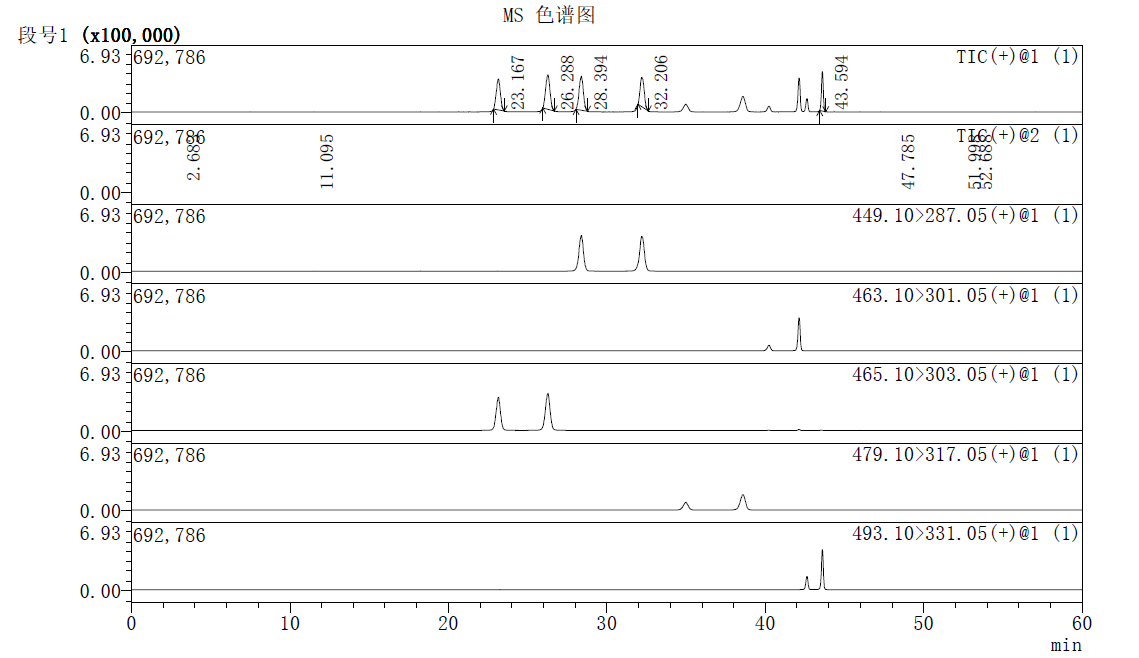


**Fig S2.** Mass spectrogram of bilberry extract

1. **Morphological observation of inhibition of cell growth of ANC**

**
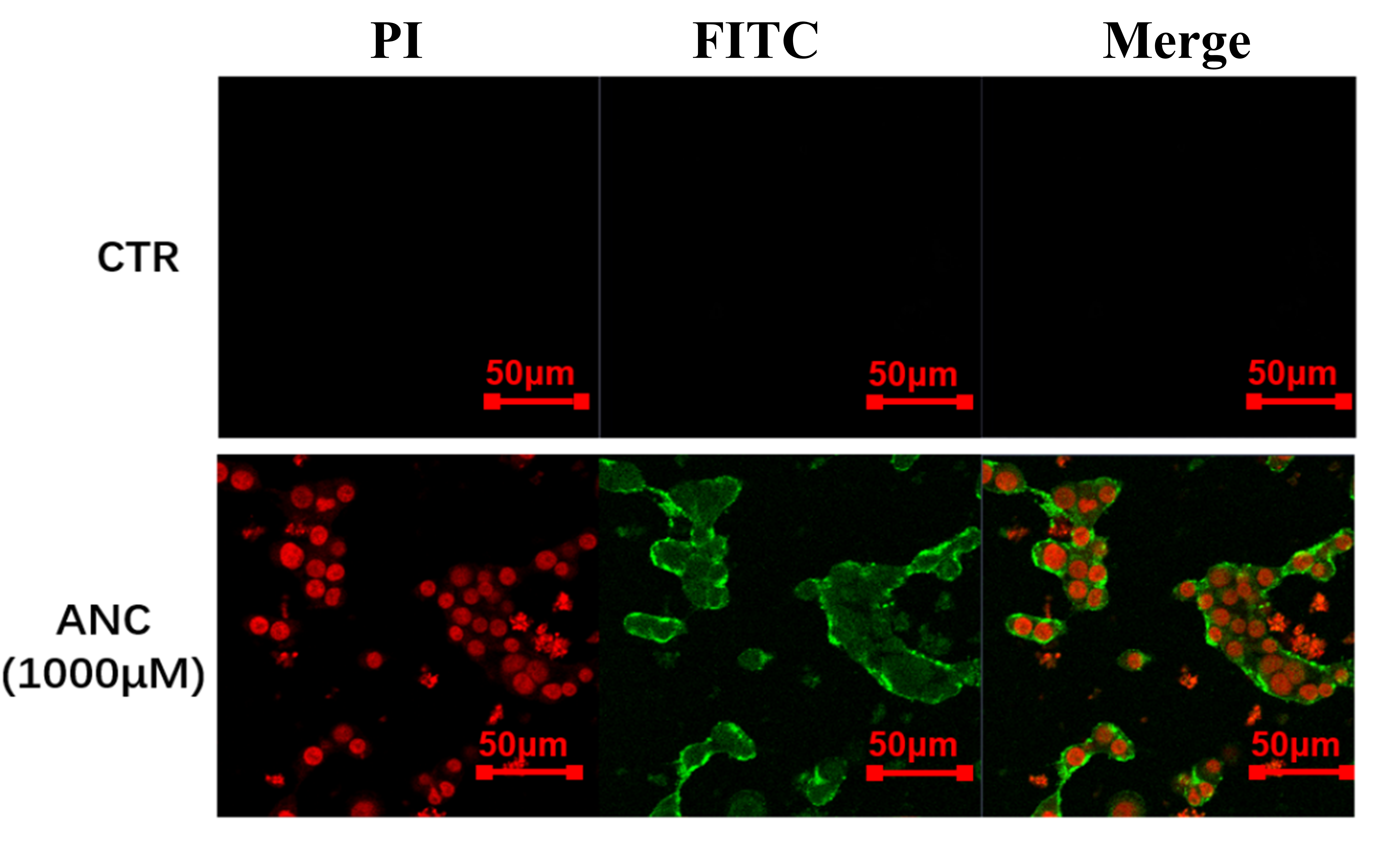
**

**Fig S2.** Laser confocal microscopy images of Annexin V-FITC/PI in MC38 cells.

1. **IC50 of different cells at different concentration of bilberry extract (mg/mL)**

**Table S2.** IC50 of different cells at different treatment time of bilberry extract (mg/mL)

| **Cell Type** | **24h** | **48h** | **72h** |
| --- | --- | --- | --- |
| MC38 | 0.84 | 0.78 | 0.45 |
| L929 | 1.20 | 1.09 | 0.78 |
